# The PWWP domain of the human oncogene WHSC1L1/NSD3 induces a metabolic shift toward fermentation

**DOI:** 10.18632/oncotarget.11253

**Published:** 2016-08-12

**Authors:** Germana B. Rona, Diego S. G. Almeida, Anderson S. Pinheiro, Elis C. A. Eleutherio

**Affiliations:** ^1^ Department of Biochemistry, Institute of Chemistry, Federal University of Rio de Janeiro, 21941-909, Rio de Janeiro, RJ, Brazil

**Keywords:** PWWP, NSD3, Pdp3, Saccharomyces cerevisiae, cancer

## Abstract

WHSC1L1/NSD3, one of the most aggressive human oncogenes, has two isoforms derived from alternative splicing. Overexpression of long or short NSD3 is capable of transforming a healthy into a cancer cell. NSD3s, the short isoform, contains only a PWWP domain, a histone methyl-lysine reader involved in epigenetic regulation of gene expression. With the aim of understanding the NSD3s PWWP domain role in tumorigenesis, we used *Saccharomyces cerevisiae* as an experimental model. We identified the yeast protein Pdp3 that contains a PWWP domain that closely resembles NSD3s PWWP. Our results indicate that the yeast protein Pdp3 and human NSD3s seem to play similar roles in energy metabolism, leading to a metabolic shift toward fermentation. The swapping domain experiments suggested that the PWWP domain of NSD3s functionally substitutes that of yeast Pdp3, whose W21 is essential for its metabolic function.

## INTRODUCTION

Emerging evidence indicates that cancer is primarily a metabolic disease involving disturbances in energy production through respiration and fermentation [1]. Even though very specific processes underlie cell malignant transformation, a large number of unspecific factors are able to initiate the disease, including radiation, chemicals, viruses, inflammation, etc. The apparent contradiction that such unspecific processes are able to cause the disease through a very specific and common mechanism was pointed out by Albert Szent-Györgyi, and considered the “oncogenic paradox” [2]. In addition, the mutation rate for most genes, including those considered essential for manifesting the hallmarks of cancer [3] is low, which makes it unlikely that the numerous pathogenic mutations found in cancer cells would occur sporadically within a normal human lifespan [4].

Although compelling evidence shows that genomic instability is present to some degree in all tumor cells [5], it is unclear how this phenotype relates to the origin of the disease. Even though no specific gene mutation or chromosomal abnormality is common to all cancers, nearly all cancers express aerobic fermentation (Warburg effect), regardless of their tissue or cellular origin [6], which is a robust metabolic hallmark of most tumors. As reviewed by Thomas N. Seyfried [1], several reports correlate respiratory dysfunction and mitochondrial structural defects to abnormalities in DNA repair mechanisms and the upregulation of fermentation pathways [7, 8], leading to carcinogenesis. This evidence supports the idea that cancer is a disease of metabolic origins. In such cases, oncogene upregulation becomes essential for increased glucose and glutamine metabolism following respiratory impairment [9]. The synthesis of nucleotides and fatty acids, as well as the consumption of glucose and glutamine are prevalent among tumor cell lines. These activities, especially glutamine intake, are used as reducing power and anaplerosis. Cancer cells metabolism is modified to simplify the uptake of nutrients into the biomass requirements to proliferation and cell growth. Recent studies show that several signaling pathways implicated in cell proliferation regulate metabolic pathways that incorporate nutrients into biosynthetic pathways. Some mutations enable cancer cells to acquire and metabolize nutrients in a way that favors proliferation rather than efficient ATP production [10, 11].

Gene amplification is a major mechanism for oncogene activation in human cancers [12–14], resulting in gene overexpression at both the RNA and protein levels [15]. Amplification of the short arm of human chromosome 8 has been reported in 10–15% of breast cancers and harbors several candidate oncogenes [12, 13, 15–17]. The 8p11-12 amplicon has been associated with estrogen receptor-positive tumors and lobular histology [18]. Recent studies have identified the Wolf-Hirschhorn syndrome candidate 1-like 1 gene (WHSC1L1, also known as NSD3) as one of the major leader oncogene candidates from the 8p11-12 region in breast cancer [15].

NSD3 is the third member of the NSD (nuclear receptor SET domain- containing) family. All proteins from this family have been directly linked to multiple human diseases. A striking feature of the three NSD proteins is that they are highly similar within a region of about 700 amino acids spanning a catalytic SET domain together with a pre and post-SET (Enhancer of zeste) domain, two PWWP (proline-tryptophan-tryptophan-proline) domains, five PHD (plant homeodomain) fingers, and a NSD-specific Cys-His rich domain (C5HCH) [19]. However, the similar domain architecture of the three NSD members does not indicate a functional redundancy [20]. NSD3 has been identified as a frequently amplified gene in breast cancer cell lines and primary breast carcinoma [21]. NSD3 has two main isoforms, NSD3l (long NSD3,1437 amino acid) and NSD3s (short NSD3, 645 amino acid) [15, 21], derived from alternative splicing of exon 10 [22]. Both NSD3 protein isoforms contain a PWWP domain; however, the short isoform presents only a single one [15]. Yang and co-workers have shown that both isoforms are located in the nucleus and might act as oncoproteins as they exhibit transforming properties [15].

The PWWP domain is exclusively found in eukaryotes, ranging from lower eukaryotes, such as protozoa and yeast, to human. The human genome encodes more than twenty PWWP-containing proteins, which are always located in the nucleus and play a major role in cell division, growth and differentiation. They are implicated in various chromatin functions, including DNA modification, repair, and transcriptional regulation [23, 24]. The PWWP domain acts as a chromatin modification reader by recognizing both DNA and histone methylated lysines at the level of the nucleosome [25, 26]. Despite the significant progress in understanding the PWWP domain function, many questions are yet to be answered, including its role in tumorigenesis.

The budding yeast *Saccharomyces cerevisiae* has been extensively used as a model for genetic analysis of various complex pathways and processes, including cell division, secretion, transcription and receptor-mediated signal transduction [27]. Due to genetic and metabolic similarities between *S. cerevisiae* and cancer cells, this microorganism has often been used as a tool for cancer research [28]. There are strong similarities between mammalian and yeast cell metabolism regulation by oncogenes/oncogene homologues. An interesting approach is the use of “tumorized yeasts” as a model for anti-cancer drug screening and for metabolism studies in order to determine how each one of these mutations would contribute to the profound metabolic alterations in cancer [29]. There are similarities between glucose catabolic repression of yeast oxidative metabolism and metabolic reprogramming of cancer cells, the Warburg effect [30]. Yeast cells growing on glucose and tumor cells show high cell proliferation and high glucose consumption rates and are both very sensitive to oxidative stress. Yeast cells engineered to express apoptosis-targeted proteins provide a powerful resource for the discovery of new genes responsible for modulation of cell-death pathways of humans and other higher organisms [27]. Like mammals, yeast undergoes apoptosis in response to oxidative stress and mitochondrial dysfunction. Escape from apoptosis and sustained cell growth are hallmarks of cancer [30–32]. Yeast is an attractive model to investigate the relations between programed cell death and mitochondrial dysfunction in both physiological and pathological conditions [33]. At least 60% of yeast genes have statistically robust human homologues or at least one conserved domain with human genes [30–32]. A substantial portion of conserved yeast and human genes perform much the same roles in both organisms, to an extent that the protein-coding DNA of a human gene can actually substitute that of yeast [27, 34]. However, if there are no direct yeast orthologous of human oncogenes/oncosuppressors, these genes can be heterologously expressed in yeast to study their function [35].

In light of these findings, the aim of this study was to evaluate the human NSD3s PWWP domain functionality in the *S. cerevisiae* experimental model.

## RESULTS

**Figure 1:**
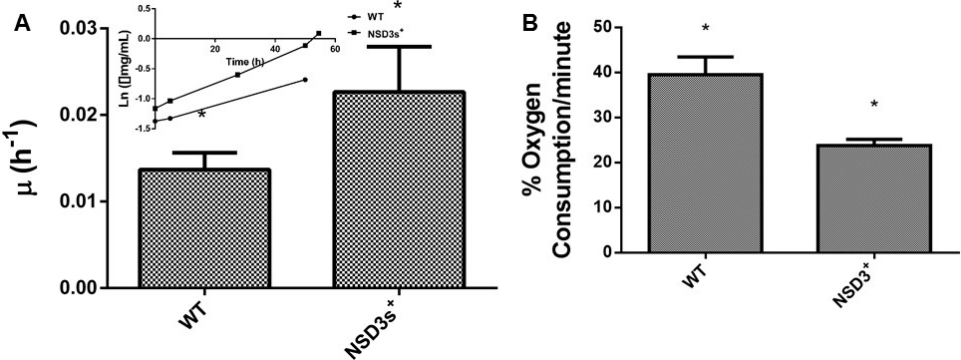
Effect of NSD3s overexpression on yeast growth rate and oxidative metabolism WT strain and the mutant overexpressing human NSD3s (NSD3s^+^ strain) were grown in drop out glycerol 4% until the middle of exponential growth phase. (**A**) For specific growth rate measurements, the absorbance at 570 nm were taken at regular intervals until cells reached stationary phase. The inset shows the growth rate. (**B**) A Clark electrode measured the oxygen consumption for ten minutes. The results represent the mean ± standard deviation of at least three independent experiments and *mean different results at WT vs NSD3s^+^ *p < 0.05.

Initially, we analyzed the effects of NSD3s overexpression on *S. cerevisae* energy metabolism, since overexpression of NSD3s was reported to transform a human healthy cell into a cell with tumorigenic characteristics [15]. Yeast strains were grown in glycerol, a carbon source that favors oxidative respiration, so the yeast metabolic phenotype could resemble that of a mammalian healthy cell. Figure 1A shows that the NSD3s^+^ yeast strains displayed a specific growth rate (μ) higher than the control (WT). In addition, NSD3s^+^ cells exhibited a decrease in oxygen consumption when compared to WT cells (Figure 1B), indicating that overexpression of NSD3s is capable of decreasing cell respiratory capacity leading to a faster proliferation.

**Figure 2:**
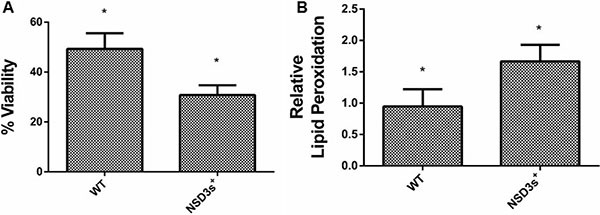
NSD3s overexpression increases yeast sensitivity to oxidative stress WT strain and the mutant overexpressing human NSD3s (NSD3s^+^ strain) were grown in drop out glycerol 4% until the middle of exponential growth phase. Oxidative damages were analyzed after stress with 20 mM H_2_O_2_/1 h/28^°^C/160 rpm. (**A**) Cellular viability was measured by standard dilution following plating on solid YPD medium for 72 h. Colony-forming units were counted and expressed as the percentage of cell viability, calculated by the (number of cells after stress/number of cells before stress) * 100. (**B**) The levels of lipid peroxidation were determined by the TBARS method and results were expressed as a ratio between the level of lipid peroxidation of stressed cells and control situation (before stress). The results represent the mean ± standard deviation of at least three independent experiments and *mean different results at WT vs NSD3s^+^ *p < 0.05.

It is well known that inhibition of the respiratory chain impairs the expression of antioxidant enzymes, which causes the cell to be more sensitive to reactive oxygen species (ROS) and results in oxidative damage [36–38]. Downregulation of oxidative metabolism occurs both in the Warburg effect (tumor cells) and during catabolic repression (yeast). In such conditions, cells do not develop an efficient antioxidant defense against ROS, which are able to damage all major cellular building blocks, including DNA, lipids and proteins. These damages can lead to cell death, accelerate the aging process and the development of age-related diseases [39, 40]. Figure 2A shows that NSD3s^+^ yeast cells were less viable than the control when submitted to oxidative stress conditions. In addition, NSD3s^+^ mutant cells showed an increase of more than 50% in the levels of lipid peroxidation after one hour of hydrogen peroxide (20 mM) exposure (Figure 2B).

To examine the heterologous expression of NSD3s in yeast, we used an anti-his tag antibody, since the construct contains an N-terminal six-histidine tag. We performed a western blotting analysis and found that the short isoform of NSD3 was successfully expressed in yeast (Supplementary Figure S1).

**Figure 3:**
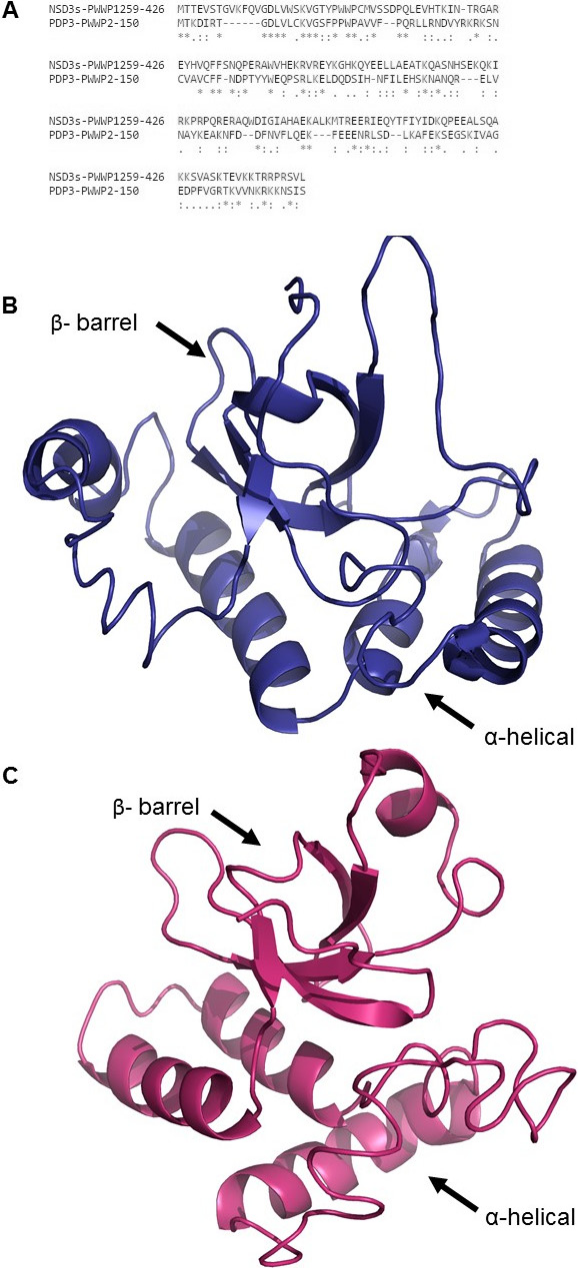
Primary sequence alignment and 3D structural models of NSD3s and Pdp3 PWWP domains (**A**) Sequence alignment between the PWWP domains of NSD3s and Pdp3. (**B**) 3D structural model of NSD3s PWWP. (**C**) 3D structural model of Pdp3 PWWP.

Next, we screened protein databases for yeast proteins containing a PWWP domain. Among them, we selected Pdp3, which has a single PWWP domain with 25% sequence identity to that of NSD3s (Figure 3A). Pdp3 is primarily located in the nucleus, but undergoes cytoplasmic shuttling in response to stress conditions such as hypoxia [41]. According to the Uniprot database [42], the PWWP domain of Pdp3 comprises amino acids 7–68 [43]; however, Gilbert and co-workers demonstrated that Pdp3 residues 1–150 are required for the recognition of histone 3 trimethylated lysine 36 (H3K36me3). These data suggested that Pdp3, and likely other PWWP-containing proteins, requires a C-terminal α-helical region for its aromatic cage stability and function [43]. We constructed a structural model for the PWWP domains of NSD3s and Pdp3 extending beyond the predicted PWWP domain (Figure 3B and 3C, respectively) using the I-Tasser server [44] for *ab initio* modelling. One can observe that both structures are composed of a N-terminal β-barrel responsible for recognizing and binding histone methylated lysines [15] and a C-terminal α-helical substructure. An alignment between the NSD3s and Pdp3 PWWP structures was performed using TM-Align [45]. TM-score was used to assess the topological similarity between the two protein structures, while root mean square deviation (RMSD) measured the average distance between the backbone atoms of the superimposed proteins. Such parameters were used to analyze the topology and the structural similarity of the models [46, 47]. The RMSD values between the Pdp3 and NSD3s PWWP models was ˜3.8Å for the full-length proteins, including the C-terminal α-helical region, and ˜2.3Å when just the PWWP domain suggested by Uniprot (UniProt #Q06188 residues 7–68 (Pdp3) and UniProt # Q9BZ95 residues 270–333 (NSD3s)) was taken into consideration. This RMSD difference is consistent with similar 3D structures [44]. The TM-score was ˜0.5 when aligning full-length Pdp3 and NSD3s PWWP domains and 0.65 when just the PWWP domain predicted by Uniprot was considered. It is known that a TM-score between 0.5˜1.0 suggests a highly similar fold [48]. Despite the low sequence similarity between the PWWP domains of NSD3s and Pdp3, our bioinformatics analysis indicated that these structures are highly similar in 3D conformation and thus possibly hold a functional relationship.

**Figure 4:**
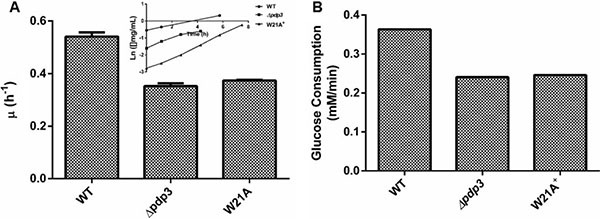
Pdp3 deficiency or W21A mutation shifts metabolism from fermentative to oxidative WT, Δ*pdp3* and W21A^+^ strains were grown in drop out glucose 2% until the middle of exponential growth phase. (**A**) For specific growth rate measurements, the absorbance at 570 nm were taken at regular intervals until cells reached stationary phase. The inset shows the growth rate. (**B**) Glucose consumption rates were calculated by determining residual glucose level over the time. The results represent the mean ± standard deviation of at least three independent experiments and * or **mean different results at WT vs Δ*pdp3* *p < 0.05 and WT vs W21A^+^ **p < 0,05.

**Figure 5:**
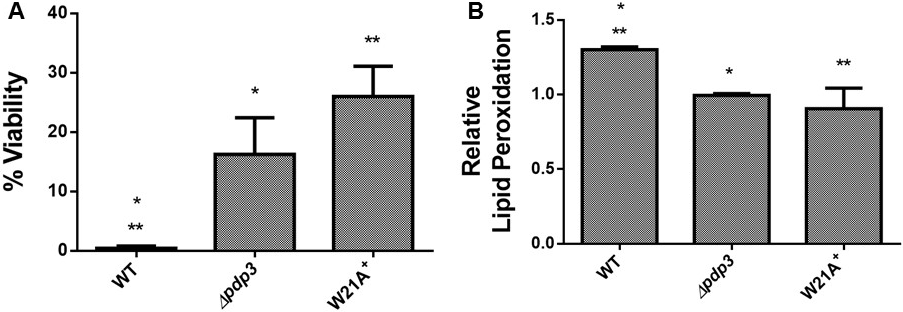
Effect of Pdp3 deficiency or W21A mutation on tolerance to oxidative stress WT, Δ*pdp3* and W21A^+^ strains were grown in drop out glucose 2% until the middle of exponential growth phase. Oxidative damages were analyzed after stress with 3 mM H_2_O_2_/1 h/28^°^C/160 rpm. (**A**) Cellular viability was measured by standard dilution plate counts and expressed as the percentage of the colony-forming units before and after stress. (**B**) The levels of lipid peroxidation were determined by the TBARS method and the results were expressed as a ratio between the level of lipid peroxidation of stressed cells and control situation (before stress). The results represent the mean ± standard deviation of at least three independent experiments and * or **mean different results at WT vs Δ*pdp3* *p < 0.05 and WT vs W21A^+^ **p < 0,05.

PWWP domains employ an aromatic cage to interact with specific trimethylated histones. The aromatic residues are conserved within the PWWP domain of Pdp3 at positions F18, W21, and F48 [43]. Therefore, we asked whether the PWWP domain aromatic cage stability was important for its metabolic function. To answer that question, we deleted the Pdp3 protein from *S. cerevisiae* (Δ*pdp3*) and transformed this mutant with a plasmid expressing Pdp3 harboring the tryptophan to alanine at position twenty one (W21A) mutation. Yeast strains were grown in glucose, a carbon source that favors fermentative metabolism, so the metabolic phenotype resembles that of a tumorigenic cell. We reasoned that both Δ*pdp3* and W21A^+^ would present similar characteristics when compared to the control. Both yeast strains showed lower specific growth rate compared to the control (Figure 4A). In addition, WT cells displayed glucose consumption rates significantly higher than the mutants (Figure 4B). In relation to ROS sensibility, both *Pdp3* deficiency and expression of the *Pdp3* W21A mutant enhanced tolerance against ROS (Figure 5). After peroxide stress, the Δ*pdp3* and W21A^+^ survival rates were higher than the WT. Moreover, lipid peroxidation levels were decreased in Δ*pdp3* and W21A^+^ compared to WT (Figure 5B).

**Figure 6:**
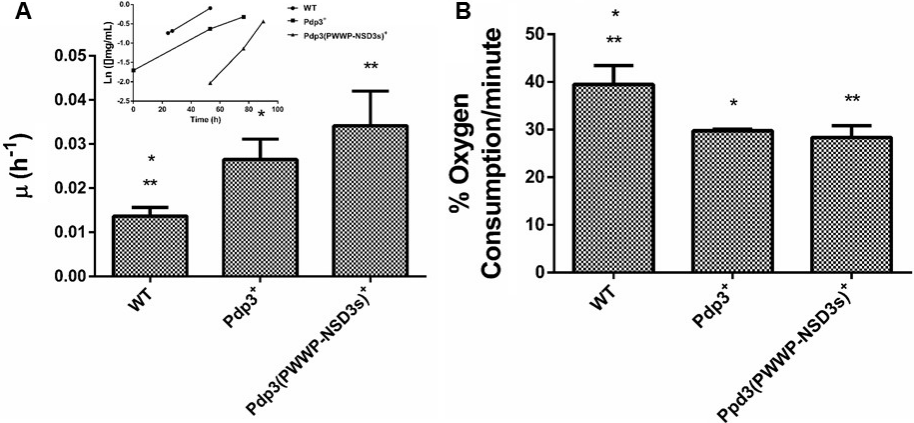
The PWWP domain of NSD3s functionally substitutes that of Pdp3 WT strain and the mutant overexpressing Pdp3 (Pdp3^+^ strain), as well as the mutant expressing the Pdp3 chimera carrying the PWWP domain of NSD3s (Pdp3 (PWWP-NSD3s)^+^) were grown in drop out glycerol 4% until the middle of exponential growth phase. (**A**) For specific growth rate measurements, the absorbance at 570 nm were taken at regular intervals until cells reached stationary phase. The inset shows the growth rate. (**B**) A Clark electrode measured the oxygen consumption for 10 min. The results represent the mean ± standard deviation of at least three independent experiments and * or **mean different results at WT vs Pdp3^+^ *p < 0.05 and WT vs Pdp3 (PWWP-NSD3s)^+^ **p < 0,05.

**Figure 7:**
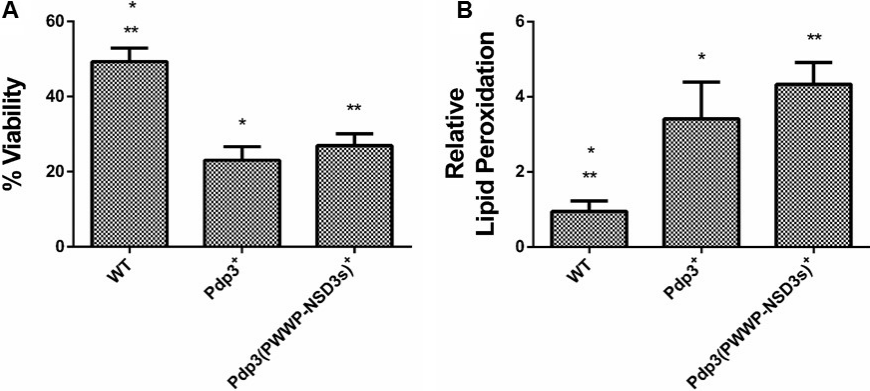
Effect of Pdp3 and Pdp3 (PWWP-NSD3s) chimera overexpression on tolerance to oxidative stress WT strain and the mutant overexpressing Pdp3 (Pdp3^+^ strain), as well as the mutant expressing the Pdp3 chimera carrying the PWWP domain of NSD3s (Pdp3 (PWWP-NSD3s)^+^) were grown in drop out glycerol 4% until the middle of exponential growth phase. Oxidative damages were analyzed after stress with 20 mM H_2_O_2_/1 h/28^°^C/160 rpm. (**A**) Cellular viability was measured by standard dilution plate counts and expressed as the percentage of the colony-forming units before and after stress. (**B**) Oxidative damages were analyzed after stress with 20 mM H_2_O_2_/1 h/28^°^C/160 rpm. Lipid peroxidation levels were determined by the TBARS method and the results were expressed as a ratio between the level of lipid peroxidation of stressed cells and control situation (before stress). The results represent the mean ± standard deviation of at least three independent experiments and *or **mean different results at WT vs PDP3^+^ *p < 0.05 and WT vs Pdp3 (PWWP-NSD3s)^+^ **p < 0,05.

**Figure 8:**
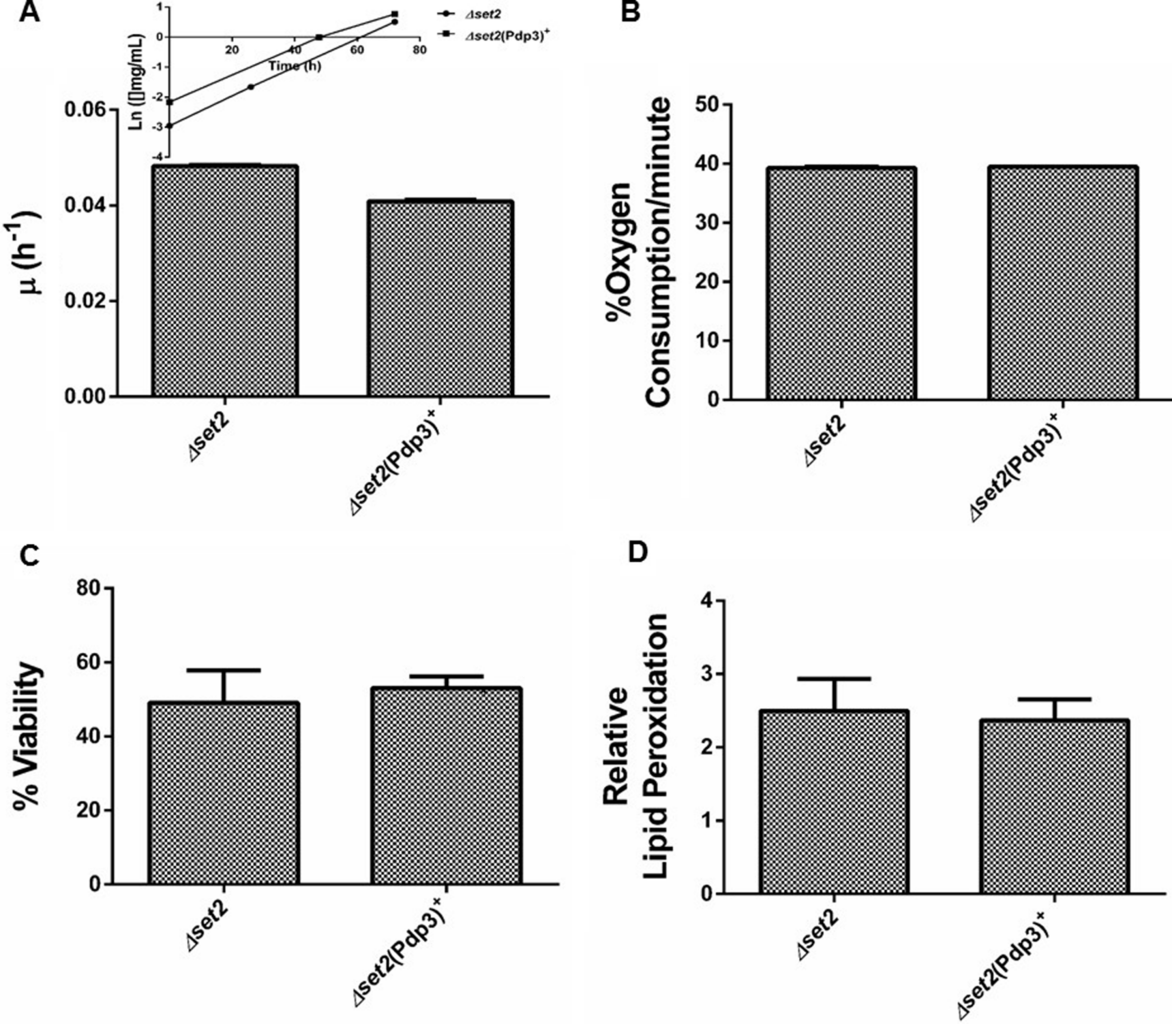
Set2 is essential for the Pdp3 recognition of H3K36me3 Δ*set2* strain and the mutant overexpressing Pdp3 (Δ*set2* (Pdp3)^+^) were grown in drop out glycerol 4% until the middle of exponential growth phase. (**A**) For specific growth rate measurements, the absorbance at 570 nm were taken at regular intervals until cells reached stationary phase. The inset shows the growth rate. (**B**) A Clark electrode measured the oxygen consumption for 10 min. Oxidative damages were analyzed after stress with 20 mM H_2_O_2_/1 h/28^°^C/160 rpm. (**C**) Cellular viability was measured by standard dilution plate counts and expressed as the percentage of the colony-forming units before and after stress. (**D**) The levels of lipid peroxidation were determined by the TBARS method and results were expressed as a ratio between the level of lipid peroxidation of stressed cells and control situation (before stress). The results represent the mean ± standard deviation of at least three independent experiments.

**Figure 9:**
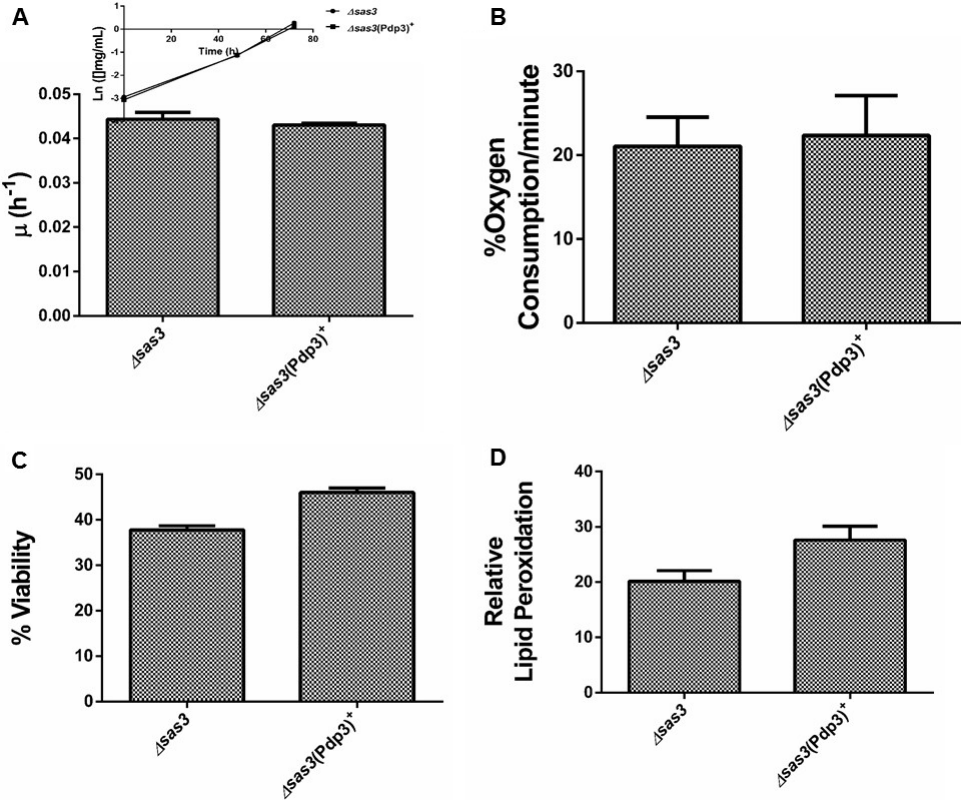
Sas3 is essential for the metabolic shift induced by Pdp3 Δ*sas3* strain and the mutant overexpressing Pdp3 (Δ*sas3* (Pdp3)^+^) were grown in drop out glycerol 4% until the middle of exponential growth phase. (**A**) For specific growth rate measurements, the absorbance at 570 nm were taken at regular intervals until cells reached stationary phase. The inset shows the growth rate. (**B**) A Clark electrode measured the oxygen consumption for 10 min. Oxidative damages were analyzed after stress with 20 mM H_2_O_2_/1 h/28^°^C/160 rpm. (**C**) Cellular viability was measured by standard dilution plate counts and expressed as the percentage of the colony-forming units before and after stress. (**D**) The levels of lipid peroxidation were determined by the TBARS method and results were expressed as a ratio between the level of lipid peroxidation of stressed cells and control situation (before stress). The results represent the mean ± standard deviation of at least three independent experiments.

Subsequently, we analyzed the effect of Pdp3 overexpression on the same metabolic phenotypes. We noticed that overexpression of Pdp3 induced an effect on cell metabolism similar to that of NSD3s. When grown in glycerol, yeast cells exhibited increased specific growth rate (Figure 6A), decreased oxygen consumption rate (Figure 6B) and higher sensitivity to ROS than the WT (Figure 7). Figure 7A shows that Pdp3^+^ yeast cells were less viable than the control when submitted to oxidative stress conditions. In addition, Pdp3^+^ mutant cells showed an increase in the levels of lipid peroxidation after one hour of hydrogen peroxide (20 mM) exposure (Figure 7B). To confirm that the PWWP domain regulates the metabolic shift through a mechanism depend on the ability of Pdp3 to bind methylated chromatin, Pdp3 was overexpressed in a *Δset2* strain. The *S. cerevisiae* Set2 protein is a histone (H3) methyltransferase highly selective for lysine 36 (H3K36) [48]. As shown in Figure 8, Pdp3 overexpression did not cause any change in the metabolic behavior of the Δset2 strain. Both Δset2 and Δ*set2*(Pdp3)^+^ strains exhibited the same growth (Figure 8A) and oxygen consumption rates (Figure 8B). In addition, tolerance to oxidative stress remained unchanged (Figure 8C and 8D). These results corroborate those obtained with the W21A^+^ mutant strain, suggesting that the effect of Pdp3 in the metabolic shift depends on the ability of its PWWP domain to recognize and bind to methylated H3K36. As previously shown, binding of Pdp3 to H3K36me3 recruits the NuA3b complex to coding regions of actively transcribed genes. Sas3, a subunit of the NuA3b complex, is a histone acetyltransferase that specifically acetylates H4, H3, and H2A [49]. However, the complete role of the NuA3b complex and the acetylation target of Sas3 remain unclear [43]. To verify if the metabolic function of Pdp3 is dependent on the NuA3b complex, Pdp3 was overexpressed in a *Δsas3* strain. Pdp3 overexpression did not lead to any change in the Δ*sas3* metabolic behavior (Figure 9), suggesting that, under respiratory conditions, the NuA3b complex is responsible for mediating the metabolic shift induced by Pdp3.

Finally, a chimeric form of Pdp3 carrying the NSD3 PWWP1 domain was constructed to analyze if the PWWP1 domain of NSD3s would be capable of functionally substituting that of Pdp3. When grown in glycerol, the chimera-expressing cells showed a higher specific growth rate (Figure 6A), a lower oxygen consumption (Figure 6B) and increased ROS sensitivity (Figure 7) than the WT, confirming that the PWWP domain of the human NSD3s protein is able to replace that of yeast Pdp3 in respiratory metabolism.

## DISCUSSION

The work presented here explores the metabolic function of the PWWP domain of the short isoform of NSD3, a human oncogene, and Pdp3, a PWWP-containing protein from *S. cerevisiae*. Using glycerol (respiratory metabolism fermentative) and glucose as the sole carbon sources we were able to mimic the metabolic profile of a cell with healthy and a tumorigenic characteristics, in order to evaluate their metabolic similarity.

Glucose is the main carbon source consumed by *S. cerevisiae*, but yeast also makes use of other carbon sources [50, 51]. Inoculation of yeast into a glucose-rich medium is followed by rapid growth driven by fermentation and subsequent production of ethanol [52]. Gene expression analysis has discovered that many genes are differentially transcribed in response to different glucose levels. A set of genes are inducible by glucose, encoding low-affinity glucose transporters, glycolytic enzymes, and ribosomal proteins. Another ensemble of genes is repressed by glucose, including those involved in utilization of alternative carbon sources, gluconeogenesis, respiration, and peroxisomal functions [50–53]. Genes involved in the glucose catabolic repression pathway are of two types: genes required for repression, such as *HXK2* and *MIG1*, and genes required for derepression, as *SNF*1 [53, 54].

Replacing glucose by glycerol as the carbon source forces yeast to obtain energy through respiration. When respiratory metabolism is adopted by yeast, glycerol is phosphorylated by glycerol kinase (Gut1) generating glycerol 3-phosphate. Then, glycerol 3-phosphate is oxidized to dihydroxyacetone phosphate (DHAP) by glycerol phosphate ubiquinone oxidoreductase (Gut2) located on the surface of the inner mitochondrial membrane. Electrons from this oxidation are transferred to ubiquinone, entering the respiratory chain [55, 56]. DHAP enters the glycolytic pathway, generating NADH (cytoplasm) and pyruvate, which goes to the respiratory chain. Glycerol-3-phosphate dehydrogenase (Gdp1/2) helps the re-oxidation of cytoplasmic NADH [57].

Healthy cells use mitochondrial oxidative phosphorylation for energy production [58], while tumor cells exhibit a significant increase in glycolysis and in the expression of glycolytic enzymes, converting most of glucose into L-lactate even under normal oxygen levels [11, 59]. This phenomenon is known as the Warburg effect and represents a metabolic hallmark of tumor cells, settling the idea that cancer is also a disease of metabolic characteristics [60]. It was shown that some cancer cells can reversibly switch between fermentation and respiration, depending on the absence or presence of glucose and environmental conditions [61]. This property represents an advantage of cancer cells *in vivo*, since it can adapt its metabolism to heterogeneous microenvironments for fast-growing conditions in malignant solid tumors [62]. In this study, we showed that NSD3s, an isoform of the human oncoprotein NSD3 [15], is capable of shifting cell metabolism from aerobic (respiration) to anaerobic (fermentation). This hypothesis was confirmed by the increase of the specific growth rate and decrease in oxygen consumption, leading to a lower tolerance against ROS in yeast. In mammalian cells, NSD3 overexpression induced the formation of expanding colonies in insulin-free medium. These cells were able to grow continuously in the absence of insulin-like growth factors, displayed the highest proliferation rate, and formed three-dimensional colonies in soft agar [15]. Based on the number of altered phenotypes acquired by NSD3-overexpressing mammalian cells, Yang and co-workers concluded that NSD3 is an important transforming oncogene from the 8p11-12 region [15]. Taken together, our results suggest that yeast cells overexpressing NSD3s display a metabolic phenotype similar to tumorigenic cells, under conditions that stimulate respiration.

**Figure 10:**
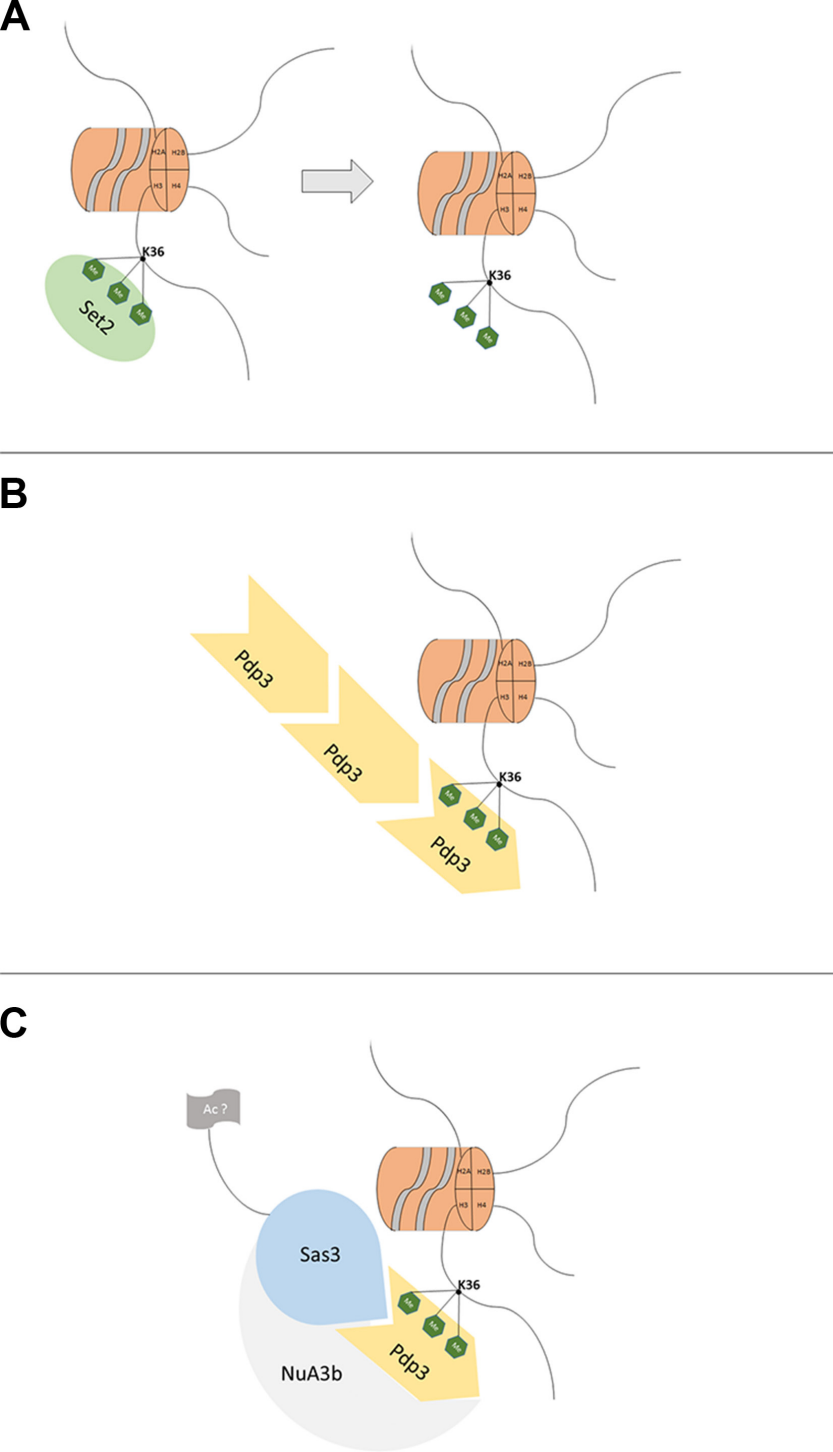
A model for the mechanism of action of Pdp3 (**A**) The Set2 histone methyltransferase trimethylates (green hexagon) histone H3 lysine 36 (black line). (**B**) Pdp3 recognizes and binds to H3K36me3. (**C**) After binding, Pdp3 recruits Sas3 and other subunits of the NuA3b complex, then Sas3 may acetylate (grey flag) histones or nonhistone proteins to facilitate transcription elongation [43].

NSD3s encodes a 645-amino acid protein containing one single PWWP domain [15, 62]. The PWWP domain is found in an extensive diversity of proteins playing a role in cell division, growth and differentiation. Several of these proteins are linked to cancer and certain diseases [63] or act as growth factors. *S. cerevisiae* harbors one PWWP-containing protein, Pdp3, which does not contain any other domain [64]. Pdp3 is a member of the NuA3 complex that shows a histone acetyltransferase activity involved in DNA transcription. The majority of genes controlled by this complex are involved in DNA replication [43]. The NuA3 complex exists in two different forms: NuA3a and NuA3b. NuA3a uses the plant homeodomain (PHD) finger of Yng1 to interact with histone 3 trimethylated lysine 4 (H3K4me3) at promoter regions of actively transcribed genes. Sas3 then acetylates histone 3 lysine 14 (H3K14), leading to transcription initiation at a subset of genes. On the other hand, Set2 trimethylates H3K36 (Figure 10A). The interaction between Pdp3 and H3K36me3 (Figure 10B), which is recognized by the PWWP domain of Pdp3 (Figure 10B), recruits Sas3 and other subunits of the NuA3b complex (Figure 10C) to the coding regions of actively transcribed genes. Although the function of NuA3b is not fully understood, Sas3 may acetylate histones or non-histone (Figure 10C) proteins to facilitate transcription elongation [43]. Pdp3 depends on the ability of its PWWP domain to bind methylated chromatin (H3K36me3) in a respiratory metabolism. Our results indicate that the metabolic phenotype induced by Pdp3 overexpression is dependent on the specific methylation of H3K36, which is accomplished by Set2. Furthermore, we demonstrated that Pdp3 also depends on the NuA3b complex, indicating that a mutation in other subunits of the complex impacts metabolic reprogramming. Overexpression of Pdp3 in null Sas3 had no effect on the metabolic phenotypes measured, when compared with Δ*sas3*. Therefore, our results suggest that, under respiratory conditions, the NuA3b complex is responsible for mediating the metabolic shift induced by Pdp3.

A conserved aromatic cage within the Pdp3 PWWP domain is required for H3K36me3 binding. The aromatic residues are conserved in the PWWP domain of Pdp3 at positions F18, W21, and F48 [43]. We found that the integrity of the hydrophobic cage is essential for the Pdp3 metabolic function. The low specific growth rate, decreased glucose consumption, and increased tolerance against ROS displayed by the W21A mutant strain can also be observed for Δ*pdp3*. These results are in accordance with those of Gilbert and co-workers that demonstrated that all three mutations (F18, W21, and F48) independently abolish the interaction between Pdp3 and a H3K36me3 peptide, suggesting that Pdp3 requires a conserved aromatic cage to bind chromatin. Since the Pdp3 W21A^+^ mutant showed the same phenotype as the Δ*pdp3* strain, we concluded that the hydrophobic cage of Pdp3 PWWP is essential for its metabolic function. While overexpression of NSD3s was able to transform a healthy into a tumorigenic cell, knockdown of NSD3 in 8p11-12–amplified breast cancer cells resulted in loss of growth and survival [15]. Therefore, the absence of Pdp3 was beneficial to cells grown in glucose (fermentative metabolism).

We then concluded that overexpression of Pdp3 was capable of inducing a shift in metabolism toward fermentation, similar to overexpression of the human oncogene NSD3. The chimeric form of Pdp3, replacing its PWWP domain by that of NSD3s, functionally substitutes the yeast protein Pdp3. These data suggested that NSD3s and Pdp3 play similar roles in energy metabolism, shifting from aerobic to anaerobic metabolism. Cluntun and co-workers demonstrated that histone acetylation is sensed by glucose flux in a dose-dependent manner, which is a possible function of the Warburg effect [65]. These results corroborate our work, since Pdp3 is part of a histone acetyltransferase complex.

Human NSD3s and yeast Pdp3 are both involved in regulation of gene expression and are reported in this work as capable of causing a metabolic shift from aerobic (respiration) to anaerobic (fermentation) metabolism. The phenotypical changes observed in the strains with different profiles of expression of NSD3s and Pdp3 in the yeast model are very similar and agree well with the metabolic shift from a healthy to a carcinogenic cell. These results indicate that the pattern of gene expression is important to the malignant transformation of cell metabolic phenotypes, in accordance with previous reports. In summary, NSD3s and Pdp3 seem to play similar roles in cell metabolism and cells overexpressing both proteins, under conditions that simulate respiration, appear to metabolically behave like a tumorigenic cell. The hydrophobic cage is essential for Pdp3 function. We suggest that the PWWP domain of NSD3s functionally substitutes that of the yeast protein Pdp3. Given that chromatin remodeling and metabolic dysfunction are gaining considerable attention in cancer studies, future researches on the PWWP domains of Pdp3 and NSD3s may contribute to the design of new anti-cancer drugs.

## MATERIALS AND METHODS

### *S. cerevisiae* strains and growth conditions

WT strain BY4741 (*MATa; his3; leu2; met15; ura3*) and its isogenic mutants Δ*pdp3*, Δ*set2 and* Δ*sas3* harboring the *PDP3, SET2 and SAS3* genes interrupted by *KanMX4* were acquired from Euroscarf, Frankfurt, Germany. Stocks of both strains were maintained on solid 2% YPD (1% yeast extract, 2% glucose, 2% peptone and 2% agar) in appropriate conditions. For null mutants, the medium also contained 0.02% geneticine. *pECUh6NSD3S* (Enzimax, USA) and p*ECUh6PDP3* (Genscript, USA) 2 μ plasmids, containing *URA3* as selectable marker and harboring the NSD3s and *PDP3* genes, respectively, under control of the *CUP1* promoter, were used to transform WT strain using the lithium acetate protocol [66]. Similarly, the Pdp3 W21A and the Pdp3 chimera carrying the PWWP domain of NSD3s, acquired from Genscript, USA, were expressed in Δ*pdp3* using the same vector. The Pdp3 (PWWP-NSD3s) chimera (DNA sequence: ATGACAACGGAAGTG TCCACTGGTGTTAAGTTTCAGGTTGGCGATCTTGT GTGGTCCAAGGTGGGAACCTATCCTTGGTGGCCTT GTATGGTTTCAAGTGATCCCCAGCTTGAGGTTCAT ACTAAAATTAACACAAGAGGTGCCCGAGAATATC ATGTCCAGTTTTTTAGCAACCAGCCAGAGAGGGC GTGGGTTCATGAAAAACGGGTACGAGAGTATAAA GGTCATAAACAGTATGAAGAATTACTGGCTGAGG CAACCAAACAAGCCAGCAATCACTCTGAGAAACA AAAGATTCGGAAACCCCGACCTCAGAGAGAACG TGCTCAGTGGGATATTGGCATTGCCCATGCAGAGA AAGCATTGAAAATGACTAGAGAAGAAAGAATAG AACAGTATACTTTTATTTACATTGATAAACAGCCTG AAGAGGCTTTATCCCAAGCAAAAAAGAGTGTTGC CTCCAAAACCGAAGTTAAAAAAACCCGACGACC AAGATCTGTGCTGATCAAAGAAGATCCGGAAGAT AACCAGAAATCAAATGAAGAAGAAAGCAAACCG AACATCAAACCGTCCAAAAAAAAGAGACCCACA GCTAATTCGGGAGGAAAATCAAACAGTGGCAATA AAAAGAAAGTTAAATTAGACTATTCCAGAAGAGT AGAAATTTCACAGTTATTTCGCCGCAGGATTCAA AGAAATCTAATCCAGAGAGAAACACCTCCTACTG AGCATGAGATCAAGGAAACTCATGAACTATTAAA TAGAATATATGAGAATTCTGACACCAAACGGCCCT TTTTTGATTTGAAGGCCCTACGCGAAAGCAAATTA CACAAGCTACTGAAAGCAATTGTTAATGATCCTGA CTTAGGCGAATTTCACCCACTTTGTAAAGAAATTT TACTGTCCTGGGCAGACCTAATCACAGAACTGAA GAAAGAAAAGTTGCAAGCGCTACCTACGCCTTGA was constructed replacing the PWWP domain of Pdp3 (residues 2-150; UniProt #Q06188) (DNA sequence: ACAAAAGATATTAGAACAGGCGATTTAGTGTTATG CAAAGTTGGCTCGTTTCCACCTTGGCCAGCTGTA GTATTTCCACAGCGTTTGCTGCGAAACGATGTATA TAGAAAGAGAAAATCCAATTGTGTTGCTGTTTGTT TTTTCAACGATCCAACTTATTATTGGGAACAACCC AGTAGATTAAAGGAGCTAGATCAAGACAGCATTC ACAATTTCATATTAGAACATAGTAAAAATGCAAAC CAAAGGGAATTGGTCAATGCTTATAAGGAAGCAA AAAATTTTGATGATTTCAACGTATTTTTACAAGAA AAGTTTGAAGAAGAAAACAGGTTAAGTGATCTAA AAGCGTTTGAGAAAAGTGAAGGTTCTAAAATCGT TGCCGGAGAAGATCCCTTTGTAGGTCGAACAAAA GTAGTGAATAAAAGAAAAAAAAATTCAATATCC) by the PWWP1 domain of NSD3s (residues 259-426;UniProt #Q9BZ95-3) (DNA sequence: ATGACAACGGAAGTGTCCACTGGTGTTAAGTTTC AGGTTGGCGATCTTGTGTGGTCCAAGGTGGGAAC CTATCCTTGGTGGCCTTGTATGGTTTCAAGTGATCC CCAGCTTGAGGTTCATACTAAAATTAACACAAGA GGTGCCCGAGAATATCATGTCCAGTTTTTTAGCAA CCAGCCAGAGAGGGCGTGGGTTCATGAAAAACG GGTACGAGAGTATAAAGGTCATAAACAGTATGAA GAATTACTGGCTGAGGCAACCAAACAAGCCAGCA ATCACTCTGAGAAACAAAAGATTCGGAAACCCCG ACCTCAGAGAGAACGTGCTCAGTGGGATATTGGC ATTGCCCATGCAGAGAAAGCATTGAAAATGACTA GAGAAGAAAGAATAGAACAGTATACTTTTATTTAC ATTGATAAACAGCCTGAAGAGGCTTTATCCCAAG CAAAAAAGAGTGTTGCCTCCAAAACCGAAGTTA AAAAAACCCGACGACCAAGATCTGTGCT). WT and Δ*pdp3* strains were also transformed with the pECUh6 vector, as a control. Transformants were selected in *drop out* medium (0.67% yeast nitrogen base without amino acids, 0.2% of *drop out* mixture and 2% agar) supplemented with 2% glucose. For all experiments, cells were grown up to mid-exponential phase (0.8 mg dry weight/ml) in liquid *drop out* medium, with or without uracil, and with 2% glucose or 4% glycerol, at 28^°^C and 160 rpm, with the ratio/flask volume medium of 5:1. The *CUP1*-regulated expression was induced with 50 μM CuSO_4_ [67].

### Bioinformatics analysis

The sequences of NSD3s (UniProt #Q9BZ95-3) and Pdp3 (UniProt #Q06188) PWWP domains were retrieved from the UniProt database [42]. The sequences were aligned using T-coffee [68]. ITasser was used for the *ab initio* modelling of the NSD3s and Pdp3 PWWP domains [44]. The TM-scores and root mean square deviations (RMSDs) of the mutant structures with respect to the wild-type structure were calculated using TM-Align [45].

### Specific growth rate (μ)

The specific growth rate (μ) during the exponential phase was determined from a linear regression fit of the semilog plot of cell growth.

### Oxygen consumption

Oxygen consumption was followed at 25^°^C in cell suspensions at 10 mg dry weight/mL incubated in 100 mM glucose and 50 mM Tris–HCl (pH 4.5) using a computer-interfaced Clark electrode operating in an air-tight chamber with continuous stirring [71, 72]. Addition of 2 mM CN^-^ completely abolished the oxygen consumption of WT cells (both when CN^-^ is added in the middle of the curve or when cells are previously incubated for 20 min), confirming that oxygen consumption was solely due to mitochondrial activity (Supplementary Figure S2). The rate of oxygen consumption was calculated from the slope of the curves. The percentage of oxygen consumption rate (%oxygen consumption/minute) was calculated from the maximum oxygen consumption trace of dithionite, which was used as a positive control (100%) and the fitting of each measurement curve. Each measurement was performed three times independently, and the oxygen consumption rate was expressed as the mean value ± standard deviation.

### Glucose consumption

Cell suspensions were centrifuged, the supernatant was discarded and the pellet was resuspended in 50 mM phosphate buffer pH 6.0, 20 mM glucose, at a final cell concentration of 3 mg dry weight/mL. Over the time, samples were collected, centrifuged and the cell-free supernatants were used for glucose consumption determination. The concentration of glucose in the supernatant was measured by HPLC (Shimadzu) equipped with a refractive index detector. An Aminex HPX-87H column (7.8 mm ID × 30 cm, BioRad, USA) was used for separation. The HPLC apparatus operated with a mobile phase of 0.004 mM sulfuric acid at a flow rate of 0.6 mL/min [71, 72].

### Oxidative stress, cell viability and lipid peroxidation

Cells at the first exponential phase growing on drop out medium glucose 2% or glycerol 4% were directly stressed (3 mM or 20 mM H_2_O_2_ [73], respectively, during 1 h at 28°C/160 rpm). Cell viability was determined by standard dilution plate counts on solid YPD medium. Viability was expressed as the percentage of colony forming units of stressed cells related to the control [69, 74, 75]. Lipid oxidation was measured by TBARS (thiobarbituric acid reactive species) method, which detects malondialdehyde, a final product of lipid peroxidation. Briefly, cells were centrifuged and washed with cold distilled water. The cell pellets were resuspended in 0.5 ml of 10% TCA (w/v) followed by addition of 1.5 g of glass beads. The samples were lysed by 6 cycles of 20 s agitation on a vortex followed by 20 s on ice. Extracts were centrifuged and the supernatant mixed with 0.1 ml of 0.1 M EDTA and 0.6 ml of 1% (w/v) thiobarbituric acid prepared in 0.05 M NaOH. The reaction mixture was incubated in a boiling water bath for 15 min and, after cooling, the absorbance was measured at 532 nm [76–78].

### Statistical analysis

Data were expressed as mean values ± SD of at least three independent experiments. Values were compared by Student's *t* -test, which denotes homogeneity between experimental groups at *p <* 0.05.

## SUPPLEMENTARY MATERIALS FIGURES


