# Supplementary material for: The PWWP domain of the human oncogene WHSC1L1/NSD3 induces a metabolic shift toward fermentation
Source: Oncotarget. 2016 Aug 12;8(33):54068–81. doi: 10.18632/oncotarget.11253 (PMC5589563; doi:10.18632/oncotarget.11253)
Supplement: Supplementary file 1 [file oncotarget-08-54068-s001.pdf]

The PWWP domain of the human oncogene WHSC1L1/NSD3 induces a metabolic shift toward fermentation

Supplementary Materials

SUPPLEMENTARY MATERIALS AND METHODS

Electrophoresis and Western blotting

For the western blot, protein concentrations were normalized using the Stickland assay. After normalization, samples were loaded on a 12% SDS-PAGE. Subsequently, samples were transferred to a nitrocellulose membrane. After transfer was completed, the membrane was blocked with 5% milk in TBS-Tween 0.1% for 2 h. The membrane was briefly washed with TBS-Tween 0.1% before the primary anti-his tag antibody was added at a 1:1,000 dilution in 3% BSA in TBS-Tween 0.1%. The primary antibody was allowed to incubate overnight and the blot was washed with TBS-Tween 0.1% afterwards. The secondary anti-rabbit antibody was added at a 1:2,000 dilution in 3% BSA in TBS-Tween 0.1% for 2 h. The blot was washed with TBS and revealed with NBT - BCIP in ALP buffer.

Selection of yeast proteins containing a PWWP domain

PWWP-containing proteins from yeast were searched in the *Saccharomyces cerevisiae* database (http://www.yeastgenome.org/). Only two proteins were retrieved from this search, Pdp3 and Ioc4. Then, a sequence alignment was performed using the T-coffe algorithm. The alignment between Ioc4 and NSD3s primary sequences showed a score of 23.38; while the alignment between Pdp3 and NSD3s had a score of 24.55. Thus, Pdp3 was chosen as the protein of work. In addition, Stec and co-workers cite the Ioc4 protein as a false positive [1]. The PWWP domains of Pdp3 and Ioc4 were modelled using the I-TASSER server. Pdp3 PWWP showed greater structural similarity to the PWWP domain of NSD3s, so it was chosen as an ortholog of this domain in yeast. The structural analysis is described in the main text of the article. The great similarity between the two models suggests a possible functional relationship and underlies our hypothesis presented in the paper.

REFERENCE

1. Stec I, Nagl SB, van Ommen GJB, den Dunnen JT. “The PWWP domain: a potential protein–protein interaction domain in nuclear proteins influencing differentiation?”. FEBS Lett. 200; 473:1–5.

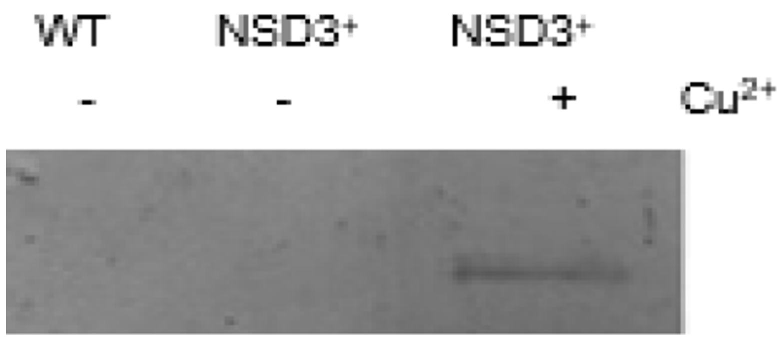

Supplementary Figure S1: Expression of NSD3s. Western blot analysis of the expression of NSD3s**.** WT strain and the mutant overexpressing human NSD3s (NSD3s^{+} strain), with or without cupper, were grown in drop out glycerol 4% until the middle of exponential growth phase.

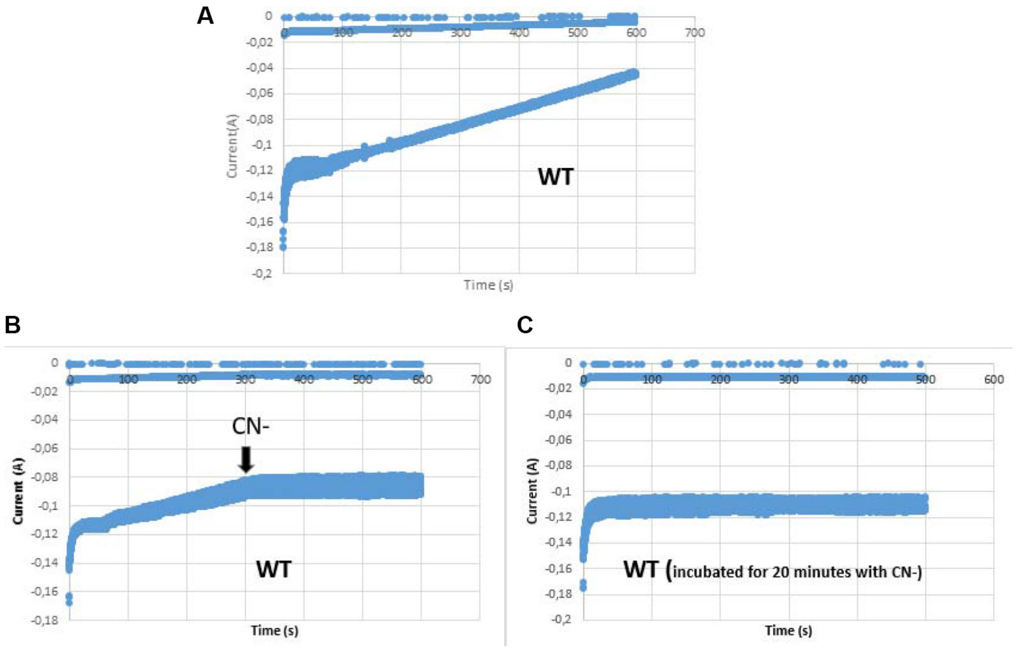

Supplementary Figure S2: Effect of cyanide in the respiratory chain. Cyanide, an inhibitor of respiratory chain, was used to confirm that we were measuring the mitochondrial oxygen consumption. The WT strain was grown in drop out glycerol 4% until the middle of exponential growth phase. A Clark electrode measured the oxygen consumption for 10 min (**A**) in the absence of cyanide (CN^-); **(B**) Addition of 20 mM CN^- after 5 min of reaction and (**C**) after a 20 min incubation.
